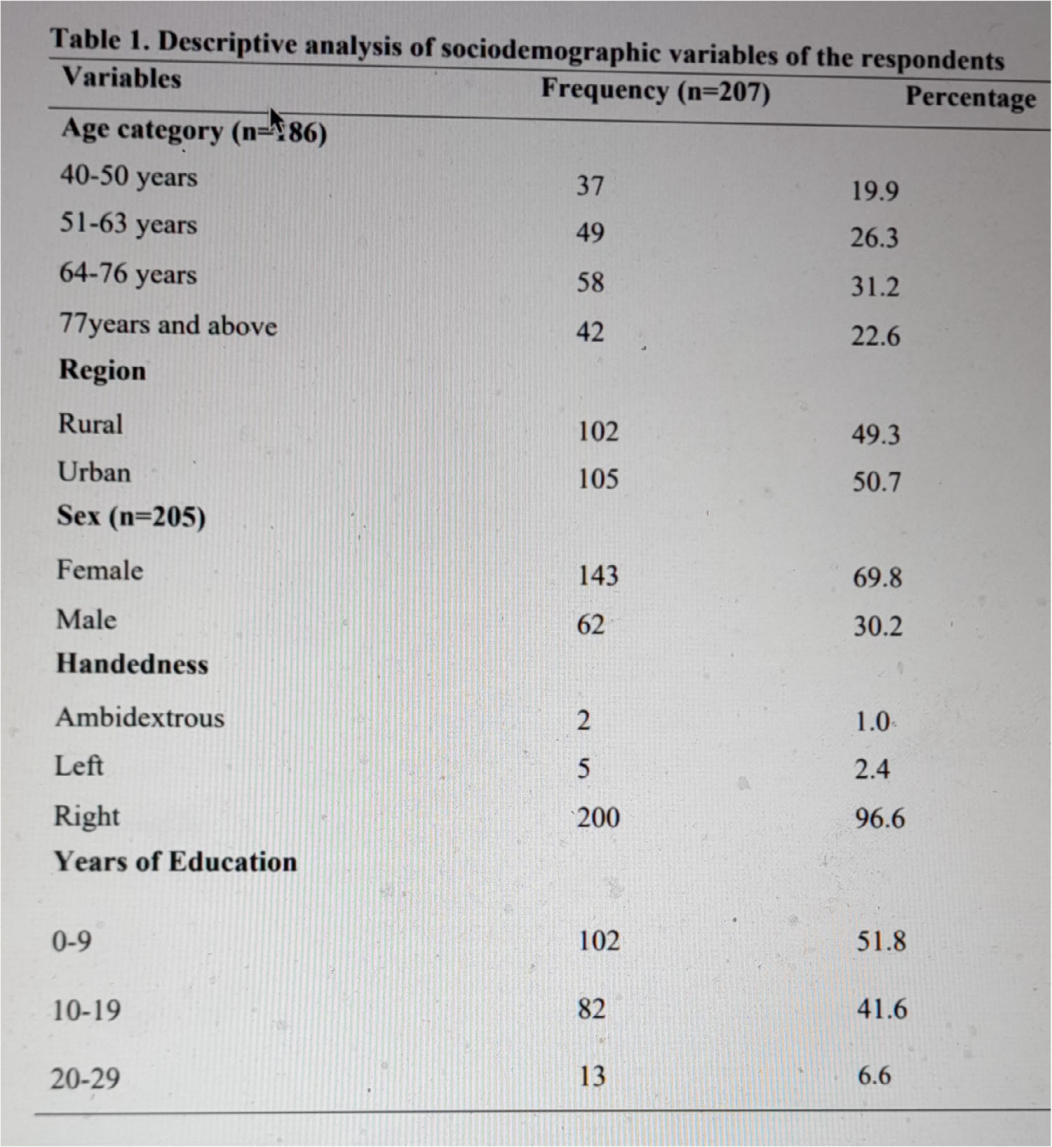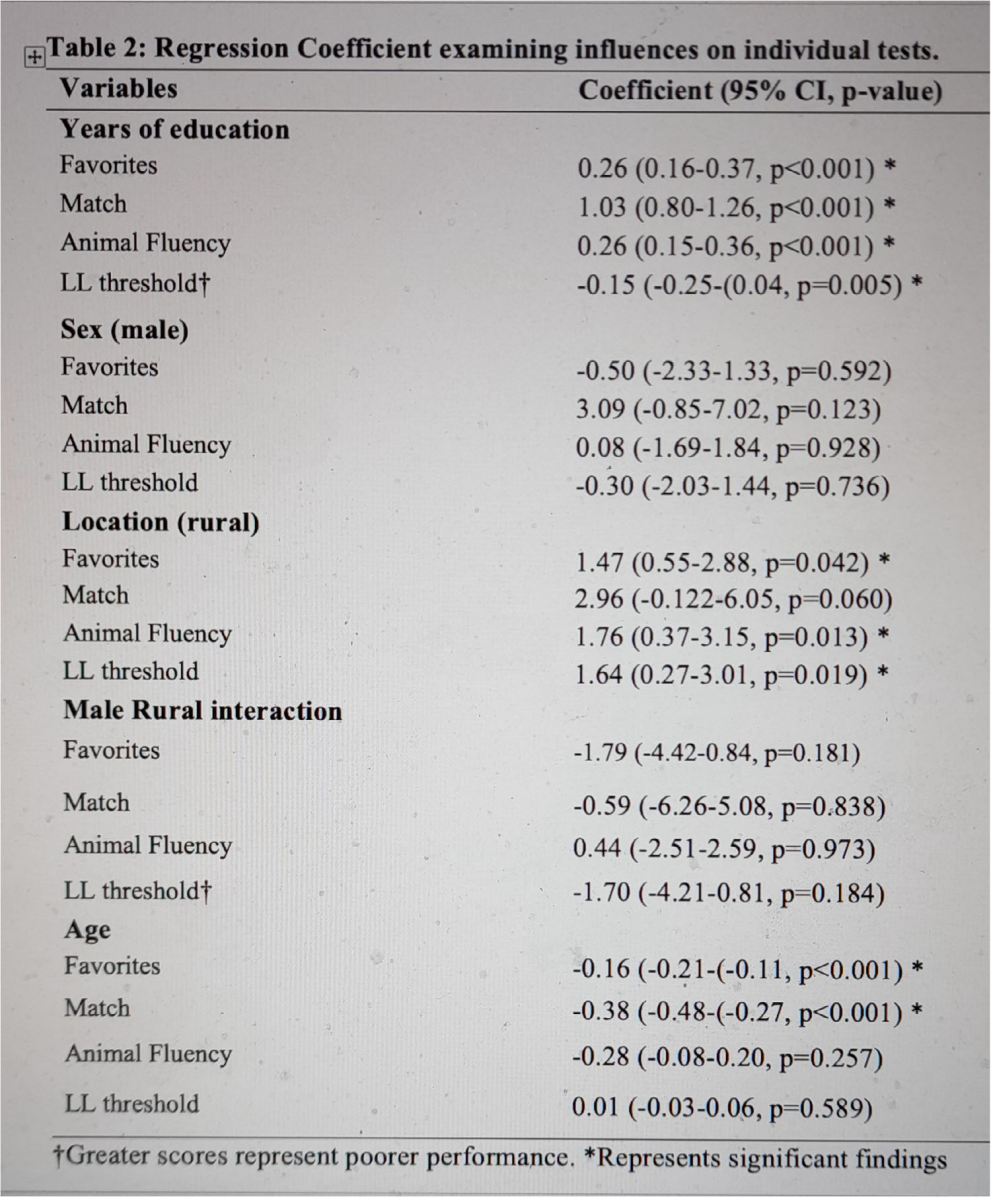# Feasibility and Determinants of Performance for a Tablet‐based Cognitive Assessment Tool in Rural and Urban Southeast Nigeria

**DOI:** 10.1002/alz70860_096447

**Published:** 2025-12-23

**Authors:** Chukwuanugo Ogbuagu, Ekenechukwu Ogbuagu, Obiageli Emelumadu, Uzoma Okereke, Irene Okeke, Godswill Chigbo, Shireen Javandel, Bruce L. Miller, Victor Valcour, Isabel Elaine Allen, Collette A Goode, Katherine L. Possin, Richard Uwakwe

**Affiliations:** ^1^ Global Brain Health Institute, University of California, San Francisco, CA, USA; ^2^ Nnamdi Azikiwe University Teaching Hospital (NAUTH), Nnewi, Nigeria; ^3^ Nnamdi Azikiwe University Teaching Hospital (NAUTH), Nnewi, Anambra, Nigeria; ^4^ School of Public Health, University of Port‐Harcourt, Port‐Harcourt, River State, Nigeria; ^5^ University of California, San Francisco, San Francisco, CA, USA; ^6^ Department of Neurology, Memory and Aging Center, University of California San Francisco, San Francisco, CA, USA; ^7^ Global Brain Health Institute, University of California, San Francisco, San Francisco, CA, USA; ^8^ Department of Epidemiology and Biostatistics, University of California, San Francisco, San Francisco, CA, USA; ^9^ Global Brain Health Institute, University of California, San Francisco, CA, USA, San Francisco, CA, USA, San Francisco, CA, USA; ^10^ Global Brain Health Institute (GBHI), University of California San Francisco (UCSF); & Trinity College Dublin, San Francisco, CA, USA

## Abstract

**Background:**

Cognitive assessment ideally is a critical element of clinical evaluations for individuals with dementia and Alzheimer's disease in Primary Health Care (PHC) settings. To examine the feasibility and demographic determinants of performance for a tablet‐based cognitive screening tool (TabCAT) battery, which includes subtests for four cognitive domains, among older PHC patients in southeast Nigeria.

**Method:**

A cross‐sectional mixed‐method descriptive study evaluating the useability and performance of TabCAT.

**Result:**

A total of 207 participants were enrolled (mean age of 64.7±13.5 years; 52% with only primary, 41% secondary, and 7% tertiary education). Most (91%) who initiated the assessment were able to complete it, requiring 10‐15 minutes to complete. More years of education was associated with better test scores across all tests (*p* < 0.001). Living in a rural location was also associated with better performance (*p* < 0.05). Male compared to female sex did not associate with performance on any of the tests (all *p*s >0.05).

**Conclusion:**

Tablet‐based cognitive assessment was feasible in rural and urban settings of Nigeria. Better performance on cognitive subtests linked to more education and residing in a rural area; however, sex did not predict performance. Digital cognitive assessment tools hold the potential for widespread use in healthcare and educational contexts, particularly in regions with varying levels of urbanization and educational access for early detection of cognitive impairments at the PHC levels.